# What is the optimal serum level for lithium in the maintenance treatment of bipolar disorder? A systematic review and recommendations from the ISBD/IGSLI Task Force on treatment with lithium

**DOI:** 10.1111/bdi.12805

**Published:** 2019-06-20

**Authors:** Willem A. Nolen, Rasmus W. Licht, Allan H. Young, Gin S. Malhi, Mauricio Tohen, Eduard Vieta, Ralph W. Kupka, Carlos Zarate, René E. Nielsen, Ross J. Baldessarini, Emanuel Severus, the ISBD/IGSLI Task Force on the treatment with lithium

**Affiliations:** ^1^ Department of Psychiatry University Medical Center Groningen, University of Groningen Groningen the Netherlands; ^2^ Aalborg University Hospital, Psychiatry Aalborg Denmark; ^3^ Department of Clinical Medicine Aalborg University Aalborg Denmark; ^4^ Department of Psychological Medicine Institute of Psychiatry, Psychology and Neuroscience, King's College London London UK; ^5^ Department of Psychiatry, Faculty of Medicine and Health, Northern Clinical School The University of Sydney Sydney NSW Australia; ^6^ Academic Department of Psychiatry Royal North Shore Hospital, Northern Sydney Local Health District, St Leonards NSW Australia; ^7^ CADE Clinic Royal North Shore Hospital, Northern Sydney Local Health District, St Leonards NSW Australia; ^8^ Department of Psychiatry & Behavioral Sciences University of New Mexico Health Sciences Center Albuquerque New Mexico; ^9^ Bipolar Disorder Program Hospital Clinic, University of Barcelona, IDIBAPS, CIBERSAM Barcelona Spain; ^10^ Department of Psychiatry Amsterdam UMC, Vrije Universteit, Amsterdam Public Health Institute Amsterdam the Netherlands; ^11^ Altrecht Institute for Mental Health Care Utrecht the Netherlands; ^12^ National Institute of Mental Health (NIMH) Bethesda Maryland; ^13^ Department of Psychiatry Harvard Medical School Boston Massachusetts; ^14^ Mailman Research Center McLean Hospital Belmont Massachusetts; ^15^ Department of Psychiatry and Psychotherapy University Hospital Carl Gustav Carus, TU Dresden Dresden Germany

**Keywords:** bipolar disorder, Lithium, maintenance treatment, serum level

## Abstract

**Aims:**

To systematically review the existing trials on optimal serum levels for lithium for maintenance treatment of bipolar disorder and to develop clinical recommendations.

**Methods:**

Systematic literature search. Discussion of major characteristics, limitations, methodological quality, and results of selected trials. Delphi survey consisting of clinical questions and corresponding statements. For statements endorsed by at least 80% of the members, consensus was considered as having been achieved.

**Results:**

With strict inclusion criteria no studies could be selected, making it difficult to formulate evidence‐based recommendations. After loosening the inclusion criteria 7 trials were selected addressing our aims at least to some extent. Four of these studies suggest better efficacy being associated with lithium serum levels in a range above a lower threshold around 0.45/0.60 and up to 0.80/1.00 mmol/L. These findings support the outcome of the Delphi survey.

**Conclusions:**

For adults with bipolar disorder there was consensus that the standard lithium serum level should be 0.60‐0.80 mmol/L with the option to reduce it to 0.40‐0.60 mmol/L in case of good response but poor tolerance or to increase it to 0.80‐1.00 mmol/L in case of insufficient response and good tolerance. For children and adolescents there was no consensus, but the majority of the members endorsed the same recommendation. For the elderly there was also no consensus, but the majority of the members endorsed a more conservative approach: usually 0.40‐0.60 mmol/L, with the option to go to maximally 0.70 or 0.80 mmol/L at ages 65‐79 years, and to maximally 0.70 mmol/L over age 80 years.

## 1 INTRODUCTION

Lithium has been licensed in Europe and North America for the long‐term maintenance treatment of bipolar disorder (BD) for more than 45 years and is justifiably considered one of the first line options in major global treatment guidelines, such as the WFSBP guideline,^1^ the NICE guideline,^2^ the RANZCP guideline,^3^ the Dutch guideline,^4^ the BAP guideline,^5^ the CINP guideline^6^ and the CANMAT/ISBD guideline.^7^ However, lithium has a very low therapeutic index, that is, a low ratio between the dose (or serum level) that is associated with toxicity (mainly CNS and renal toxicity) and the dose associated with therapeutic effect. The therapeutic index for lithium is approximately 2.^8^

For maintenance treatment of BD several reviews recommend differing *minimum* effective lithium serum levels ranging from 0.40 mmol/L^9^, ^10^, ^11^ and 0.50 mmol/L,^12^ or 0.60 mmol/L 13 to as high as 0.80 mmol/L.^14^, ^15^ In addition, some major clinical practice guidelines recommend *optimal ranges*, but these also lack consistency. For instance, the NICE guideline,^2^ the RANZCP guideline 3, 16 and the BAP guideline 5 recommend 0.60‐0.80 mmol, the CANMAT/ISBD guideline 7 0.60‐1.00 mmol/L, and the CINP guideline 6 0.60‐1.20 mmol/L, whereas other guidelines, for example, the WFSBP guideline,^1^ do not provide specific recommendations. Finally, some reviews more specifically recommend targeting serum levels for individual patients “*on the basis of efficacy and tolerability”* without further specification^15^ or “*primarily at 0.60‐0.75/0.80 guided by clinical response and tolerability”* 16 within a broad range of 0.40‐1.20 mmol/L,^10^ which is also recommended in the Dutch guideline.^4^ The major problem underlying these different recommendations is that large studies, well designed to assess the optimal serum level in maintenance treatment, specifically the serum level that best balances efficacy and tolerability for most patients, are lacking.

In clinical pharmacology recommendations for the optimal dose (or serum level) of a drug should be based on studies in which patients are randomized to different groups with various a priori defined fixed doses/serum levels (or narrow dose/serum level ranges) of the drug in question. This is necessary as in studies with flexible doses/serum levels or with wide dose/serum level ranges, prescribers are inclined to increase the dose/serum level especially in those patients who are not responding to (and are tolerating) the original dose/serum level, resulting in a so called channeling effect: nonresponding patients are treated with higher doses/serum levels than patients who have already responded to lower doses/serum levels^17^ or dosing following outcome rather than outcome following dose.^18^

To our knowledge, the most recent systematic review on optimal serum levels for lithium in the maintenance treatment of BD by Severus et al^10^ identified only 5 studies that addressed this issue, most with small samples sizes. A further limitation of this review is that several important methodological aspects and limitations of the studies were discussed only briefly. Moreover, many clinical questions have not been systematically addressed in the literature, for example, what is the best timing of blood sampling? Should the recommendations be different for dosing once daily vs twice daily? Should the recommendations be different for immediate release vs extended release formulations? Should the optimal serum level be different for the prevention of manic (and mixed) episodes compared to depressive episodes? Should recommendations take ethnicity/race or gender into consideration? And should recommendations for children and adolescents or for elderly patients be different from the recommendations for nonelderly adults?

Based on the need for an updated and more critical systematic review of the literature on optimal serum levels for lithium in the maintenance treatment of BD, a joint International Society for Bipolar Disorders (ISBD) and International Study Group on Lithium (IGSLI) Task Force on the Treatment with Lithium was charged to address this topic (and other lithium related topics). In addition, it was decided to develop a consensus on recommendations for optimal serum levels that also attempted to answer key clinical questions such as those outlined above, for which there apparently was no direct evidence available, using the Delphi method.

## 2 METHOD

### 2.1 Composition of the task force

The ISBD/IGSLI Task Force on Lithium (further Task Force) was comprised of global experts active in ISBD or IGSLI and furthermore selected on their contributions to the literature on lithium. The Task Force started at the annual conference of the ISBD in Seoul (2014) while a subgroup of the Task Force (WN, ES, RL, GM, AY, MT) initiated this project on optimal serum levels for lithium at the annual ISBD conference in Washington (2017) and further discussed it at the annual ISBD conference in Mexico City (2018).

### 2.2 Selection of literature

As a first step 2 members of the Task Force (WN and ES on August 28, 2017) initiated a systematic literature search in PubMed with the following search terms limited to “clinical trials” and “reviews”: “lithium” and “bipolar disorder” and [“serum level” or “serum concentration” or “plasma level” or “plasma concentration” or “blood level” or “blood concentration” or “lithium level” or “lithium concentration”].

From the retrieved trials we initially intended to examine and discuss only those which fulfilled a priori defined inclusion criteria (see Table 1). The included trials were rated for their methodological quality regarding randomization, blinding and description of withdrawals, and dropouts ensuring intent to treat analysis, according to the Jadad Scale^19^ as poor (score 0‐2), moderate (score 3‐4), or good (score 5).

**Table 1:** Overview of original inclusion criteria (see method) and modified inclusion criteria (see results)

#	Original inclusion criteria	Modified inclusion criteria
1	Patients with BD, either type 1 or type 2 or both or unspecified. In instances where the trial also included other patient groups, the results of BD patients had to have been reported separately, or at least 80% of the total group should comprise BD patients	Also allowing studies with patients with recurrent mood disorder, with at least some specified outcome data on patients with BD
2	Patients to be currently in remission	Also allowing studies with patients not in remission
3	Maintenance treatment with lithium aimed at preventing a new (treatment emergent) episode	Also allowing studies with lithium being used in the long‐term treatment, ie in studies with a follow‐up of at least 3 months
4	Evaluated the efficacy of lithium as monotherapy	Also allowing studies with lithium given in combination with other psychotropic drugs
5	Had a randomized controlled design	Also allowing non‐randomized controlled studies under the condition of no clear indication of channeling bias
6	Patients were assigned to groups with different treatment regimens with lithium resulting in different a priori defined fixed lithium serum level ranges	Also allowing studies comparing different fixed lithium serum level ranges, but not a priori defined

### 2.3 Delphi method

Anticipating that our literature review would fall short of providing clear evidence‐based conclusions and definite guidance regarding optimal lithium serum levels, we concurrently developed a survey consisting of clinical questions and corresponding statements using the Delphi method.^20^ Informed by the findings of clinical trials that have been conducted and by existing reviews and guidelines we formulated 32 statements on 16 clinical questions (see Supplemental Table S1) presented to all 50 Task Force members, representing 15 countries. They were asked to score all statements as “Agree” or “Not agree”, with the option in several statements to mark a preferred additional more specified response. For statements endorsed by at least 80% of the participating members consensus was considered as having been achieved.

Two survey rounds were initially planned. In the first survey round we tested the statements and besides asking members to choose among the additional responses, we also asked for comments on the statements and on the additional responses. After having received the responses and comments it was concluded that a second survey round was not needed as the comments received did not reveal a need to change statements or responses.

### 2.4 Further procedure

The subgroup that initiated this project consisted of WN, ES, RL, GM, AY, and MT. Selection of studies and data extraction was conducted by WN and ES. The first drafts of the paper and the Delphi statements were written by WN in collaboration with ES and RL and then circulated for comments and suggestions to GM, AY, and MT resulting in a second draft that was sent to all Task Force members for comments and/or discussion at (or prior to) the annual ISBD conference in Mexico City (2018). This resulted in a third draft of the paper (including introduction, the method section, and results from the systematic review but still without the results of the Delphi survey and the discussion section) that was developed in the same way and again send to all members together with the first round of the Delphi survey.

With the results of the Delphi survey a fourth complete draft (now also with the discussion section) was again written by WN, ES, RL, GM, AY, and MT, and then sent to all Task Force members for comments and approval. As last step the final version was send for final approval to all authors (now also including EV, RK, CZ, RN, and RB), while those Task Force members who at least had completed the Delphi survey and approved the manuscript were listed as members of the Task Force.

After having received the reviewers’ comments, the definite text was written by WN, ES, RL, GM, AY, and MT.

## 3 RESULTS

### 3.1 Selection of literature

**Figure 1:**
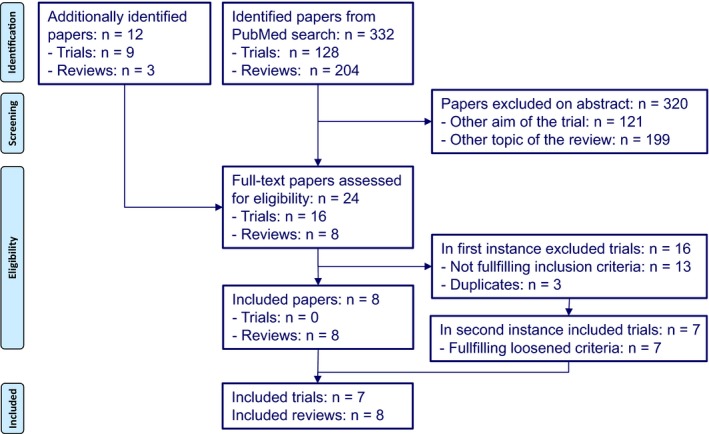
Prisma flow diagram of the selection of papers [Colour figure can be viewed at wileyonlinelibrary.com]

The results of our literature search and the further selection is presented in Figure 1.

The literature search in PubMed resulted in 128 trials and 204 reviews, of which we based on the abstracts selected 7 trials and 5 reviews for full‐text reading. In addition, we added another 12 papers from reference lists of the selected papers and already known to us: 9 trials and 3 reviews.

In the first instance we excluded all 16 selected trials, as none of the trials fulfilled all 6 inclusion criteria (see above). However, we then decided to modify the inclusion criteria by loosening them (see again Table 1).

After this adjustment 7 trials could be included; in chronological order: Jerram & McDonald^21^ (further Jerram); Waters et al^22^ (further Waters); Coppen et al^23^ (further Coppen); Maj et al^24^ (further Maj); Gelenberg et al^25^ (further Gelenberg); Vestergaard et al^26^ (further Vestergaard); and Nolen & Weisler^27^ (further Nolen). Nine other trials remained excluded on the following basis: because of possible channeling bias,^28^, ^29^, ^30^, ^31^ reporting duplicate information,^32^, ^33^ post‐hoc analyses of original studies with potential channeling bias^34^, ^35^ or addressing a different question.^36^

In addition, 8 literature reviews were selected: Sashidharan^9^; Hopkins & Gelenberg^13^; McIntyre et al^14^; Sproule^15^; Severus et al^37^; Severus et al^10^; Wijeratne & Draper^12^; and Dols et al.^38^

### 3.2 Description of the included trials

*Jerram* 21 included “*just over 80*” patients with bipolar (BP) or unipolar depressive (UP) disorder, of whom 51 BP (and 22 UP) patients completed the one year study, indicating that about 10 BP or UP patients dropped out. At baseline, all patients were in remission and using lithium while potential use of other psychotropic medication is not reported. Patients were randomized to 3 different a priori defined 12‐hour lithium level ranges: <0.50; 0.50‐0.69, and ≥0.70 mmol/L. Endpoint was “*need for additional psychotropic medication and/or admission”*.

Thus, this study fulfilled 4 of the 6 original inclusion criteria (2, 3, 5, and 6). Other limitations are that the minimum and maximum lithium levels were not defined for the lower and upper lithium level ranges respectively, that the randomization procedure is unclear, that the study was not blind, that blood was collected between 12 and 16 hours after the last intake and that only the number of BP patients who completed the study is presented, leaving unclear how many BP patients were included and how many dropped out.

*Waters* 22 included 36 BP patients of who 29 completed the 1 year study. It is uncertain whether patients were in remission, as they “*had to have reported residual endogenous mood swings which had not required hospitalization”* in the past 2 years, indicating that this study was not a pure recurrence prevention study. At baseline randomization all were using lithium while potential use of other psychotropic medication is not reported. Patients were randomized to 2 different a priori defined lithium level ranges: 0.30‐0.80 and 0.80‐1.40 mmol/L. Endpoint was “*change in mood of a sufficient degree to require clinically additional pharmacological intervention […]”*.

Thus, this study fulfilled only 3 of the original inclusion criteria (1, 5, and 6). Further limitations are that the timing of blood collection was not reported and that the procedure used for randomization is unclear.

*Coppen* 23 included 88 BP and UP patients of whom 72 completed the 1 year study, including 23 BP patients. Prior to the trial all patients already were using lithium with a mean serum level of 0.86 ± 0.20 mmol/L, and an unreported number were also using antipsychotics or antidepressants. The patients were probably also not in remission as the report presents pre‐trial morbidity data which were compared with data during the trial, implying that it was not a pure recurrence prevention study. At baseline patients were randomized to continue lithium at the same dose or to receive a dose reduction in about 25% or 50% resulting in 3 different 12‐hour lithium level ranges of 0.45‐0.59, 0.60‐0.79, and ≥0.80 mmol/L. Outcome measure was not a relapse or recurrence but the change in score on the *Affective Morbidity Index* during the trial compared to pre‐trial ratings.

Thus, this study only fulfilled 2 of the original inclusion criteria (5 and 6). Further limitations are that only the number of BP patients who completed the study is presented, leaving unclear how many BP patients were included and how many dropped out and that the randomization procedure is unclear.

*Maj* 24 studied 80 BP patients (69 completers) with recurrent episodes (at least one episode during the 2 years “*preceding the index episode and the commencement of lithium prophylaxis”*), indicating that patients were not in remission (implying that it was not a pure recurrence prevention study), but also leaving unclear whether or not patients were already using lithium prior to the trial. At baseline, patients were divided (but not randomized, personal information to ES) to 4 different groups (n = 20) with 12‐hour lithium levels ranges of 0.30‐0.45, 0.46‐0.60, 0.61‐0.75, and 0.76‐0.90 mmol/L, while potential use of other psychotropic medication is not reported. Major endpoint was “*mean number of episodes and mean total morbidity (expressed in months)”* during the 2 years of the trial compared with the 2 years prior to the trial.

Thus, this study only fulfilled 2 of the original inclusion criteria (1 and 6). Another limitation is that the study was not blind.

*Gelenberg* 25 studied 94 BP‐1 patients of whom 59 completed the 3‐year trial. All patients had to be stable for at least 2 months since the last episode and also at least 2 months tolerating lithium at serum level 0.60‐1.00 mmol/L, while potential use of other psychotropic medication at start or during the trial is not reported. At baseline patient was randomized to 2 different a priori defined lithium level ranges: 0.40‐0.60 and 0.80‐1.00 mmol/L. Endpoint was a recurrence of mania or major depression, defined by DSM‐III or RDC criteria.

Thus, this study fulfilled 5 of the original inclusion criteria (1, 2, 3, 5, and 6). A source of bias could be the abrupt lowering of the lithium levels at randomization in patients previously responding to high levels resulting in a risk of drug‐discontinuation related morbidity.^34^ Another limitation is that only the patients but not the investigators/clinicians were blind to the dosage‐assignment.

*Vestergaard* 26 included 57 BP and 34 UP patients in a 2‐year follow‐up trial. At baseline all patients were still hospitalized (indicating that they were not in remission and implying that it was not a real recurrence prevention study) and about half of them were still using antipsychotics or antidepressants. Whether (some) patients were also already using lithium remains unclear in the report. At baseline patients were randomized double blind to 2 different a priori defined lithium level ranges of 0.50‐0.80 and 0.80‐1.00 mmol/L. Endpoint was recurrence (or relapse) defined as “*re‐emergence of affective disorder of a severity that rendered re‐hospitalization necessary”*.

Thus, this study fulfilled only 2 of the original inclusion criteria (5 and 6). Further limitations are that the study was not blind and that only the number of BP patients who were included is presented, leaving unclear how many BP patients completed the study and how many dropped out.

*Nolen* 27 published a post‐hoc analysis of a long‐term study intended to test the efficacy of quetiapine.^39^ In this study patients with BP‐1 disorder with a recent manic of depressive episode who had achieved remission during ≥4 weeks of open‐label treatment with quetiapine monotherapy were randomized to 2‐years treatment of continued quetiapine or switch to placebo or lithium. According to the protocol, the 418 patients in the lithium arm should have received a lithium dose resulting in a 12‐hour lithium level of 0.60‐1.20 mmol/L, guided by a “*programmed automatic system”* to alert treating clinicians to adjust dose while maintain blind to the drug (lithium, quetiapine or placebo). However, in 54 patients no blood levels were measured while in 137 patients mean lithium levels remained below the minimum predefined level of 0.60 mmol/L, probably due to a failure of the programmed automatic system to adjust the dose, rather than due to adverse events or early response (being the most important indicators of channeling). This circumstance created the possibility to compare these 137 patients with the 201 patients receiving lithium within the predefined range of 0.60‐1.20 mmol/L and with the patients receiving placebo (n = 404). Endpoint was a recurrence defined as the “*initiation of [psychotropic medication] to treat a mood event, hospitalization for a mood event, or a YMRS or MADRS score of ≥20”*.

Thus, this study fulfilled 4 of the original inclusion criteria (1, 2, 3, and 4). Besides that the composition of both lithium arms was not the result of randomization and the levels were not a priori defined, another major limitation of this study is that it was discontinued prematurely by the sponsor after an interim analysis after 300 mood events had revealed superiority of quetiapine over placebo, which resulted in loss of probably 15%‐20% of patients before completing the 2‐year follow‐up. Finally, although called a “trough” level, it is unclear form the paper whether blood was sampled after 12 ± 1 hour after the last evening intake.

In summary, none of the 7 trial studies described above fulfilled all original inclusion criteria (Table 2). Four studies (Waters, Maj, Gelenberg, Nolen) addressed only BP patients (original criterion 1) and the remaining 3 studies (Jerram, Coppen, Vestergaard) included both BP and UP patients without a complete description of the results in de BP patients. Three studies (Jerram, Gelenberg, Nolen) included only patients who were in remission (original criterion 2) and so were pure recurrence prevention studies (original criterion 3), whilst the other 4 studies included patients with *“residual mood swings”* (Waters), who were hospitalized (Vestergaard) or included patients with morbidity (Coppen, Maj) and thus also examined the direct/acute effects. Only 1 study (Nolen) investigated solely the effect of lithium as monotherapy (original criterion 4), 2 studies (Coppen, Vestergaard) reported that at least some patients used antipsychotics and/or antidepressants, and 4 studies (Jerram, Waters, Maj, Gelenberg) did not provide information on (possible) use of concurrent medication. Five studies (Jerram, Waters, Coppen, Gelenberg, Vestergaard) were randomized (original criterion 5), implying that lithium level ranges were not affected by channeling, while the remaining 2 studies (Maj, Nolen) were not randomized, but it is likely that the composition of the different lithium groups in these studies was not affected by channeling (see also below). Finally, 6 studies (Jerram, Waters, Coppen, Maj, Gelenberg, Vestergaard) compared a priori defined lithium level ranges (original criterion 6), while the seventh study (Nolen) involved 2 groups with lithium level ranges defined post‐hoc, as well as a group receiving placebo.

**Table 2:** Original inclusion criteria met by the included studies

Inclusion criterion	1			2	3	4	5	6	
Study (year)	N Included	N BP randomized	N BP completed	Remission at randomization	Recurrence prevention	Lithium monotherapy	Randomization	A priori defined lithium levels	N inclusion criteria met
Jerram & McDonald (1978)	BP and UP "over 80"	?	51	Yes	Yes	No info	Yes	Yes	4
Waters et al (1982)	BP 36	36	29	"residual mood swings"	No	No info	Yes	Yes	3
Coppen et al (1983)	BP and UP 88	?	23	No info	No ?	Total group: APs and ADs (% no info)	Yes	Yes	2
Maj et al (1986)	BP 80	80	69	No info	No ?	No info	No	Yes	2
Gelenberg et al (1989)	BP‐1 157	94	56	Yes	Yes	No info	Yes	Yes	5
Vestergaard et al (1998)	BP and UP 91	57	???	Still hospitalized	No	Total group: APs 35%, ADs 30%, Both 14%	Yes	Yes	2
Nolen & Weisler (2012)	BP‐1 742	742	295	Yes	Yes	Only zolpidem, benzo's, and chloralhydrate	No	Indirectly yes ?	4

Abbreviations: APs, antipsychotics; ADs, antidepressants; BP, bipolar disorder; UP, unipolar depressive disorder.

Note: Green = Criterion met; Orange = Unclear; Red = Criterion not met [Colour Table can be viewed at wileyonlinelibrary.com]

Table 3 provides information on prior treatment with lithium; treatment at randomization; details regarding treatment with lithium; and treatment with other psychotropic medication at follow‐up. No study provided adequate information on all potentially relevant points. It illustrates the heterogeneity of the included studies.

**Table 3:** Treatment characteristics prior to the study, at randomization/start of the study and during follow‐up

Study (year)	Prior use ofof lithium	Medication at randomization		Lithium during follow‐up				Other psychotropics during follow‐up
		Lithium	Other medication	Abrupt changeof dose	Immediate or slow Release	N doses/day	Sampling time	
Jerram & McDonald (1978)	All	Yes	? (no info)	?	?	?	12‐16 hrs	?
Waters et al (1982)	All	Yes	? (no info)	?	?	?	?	?
Coppen et al (1983)	All	Yes	Total group: APs and ADs (% no info)	?	Slow	1 dd	±12 hrs	Total group: APs and ADs (% no info)
Maj et al (1986)	Probably not (no info)	? (no info)	? (no info)	?	Immediate ? (conventional formulation)	?	±12 hrs	?
Gelenberg et al (1989)	All	Yes	? (no info)	Abrupt	?	?	±12 hrs	?
Vestergaard et al (1998)	? (no info)	? (no info)	? (no info)	NA ? (no info)	Slow	2 dd	±12 hrs	Total group: APs 35%, ADs 30%, Both 14%
Nolen & Weisler (2012)	Probably Yes (N unknown)	None	Quetiapine	NA	?	2 dd	"Troughlevel"	Only zolpidem, benzo's, and chloralhydrate

Abbreviations: APs, antipsychotics; ADs, antidepressants; NA, not applicable.

### 3.3 Quality assessment of the included studies

Regarding JADAD criteria to rate methodological quality, 5 of the 7 studies involved randomized assignment to particular serum concentrations of lithium. However, the randomization was described adequately in only studies (Gelenberg, Vestergaard) and not in the remaining 3 studies (Jerram, Waters, Coppen); 2 studies were not randomized (Maj, Nolen). Three studies had a double‐blind design, which was well described in 1 study (Nolen) but not in the other 2 studies (Waters, Coppen); 1 study was blind for patients but not for investigators/clinicians (Gelenberg); 3 studies were open (Jerram, Maj, Vestergaard). Finally, 4 studies adequately reported the number and reasons for drop‐outs (Waters, Maj, Gelenberg, and Nolen), but the remaining 3 studies did not (Jerram, Coppen, Vestergaard).

Consequently, the JADAD quality scores for the included studies varied from low, that is, score 1 (Jerram, Maj) or 2 (Waters, Coppen, Vestergaard) to moderate, that is, score 3 (Gelenberg, Nolen). No study received score 4 or the highest score 5.

### 3.4 Clinical outcomes of the included studies

Table 4 summarizes the most important clinical outcomes of the included studies. Significant differences between lithium serum level ranges are indicated by green (better outcomes) vs red (poorer outcomes), while outcomes which were not significantly different are indicated by blue.

**Table 4:**
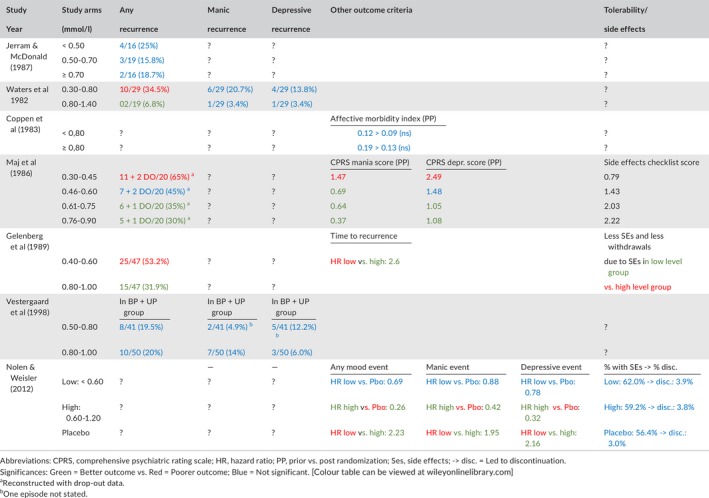
Summary of major clinical outcomes

The findings of the 7 studies are summarized in Table 4. Taken together, 4 studies (Waters, Maj, Gelenberg, and Nolen) indicate better long‐term efficacy in the prevention of any mood episode with mean lithium serum levels in a range above a lower threshold around 0.45/0.60 and up to 0.80/1.00 mmol/L. The other 3 studies (Jerram, Coppen, Vestergaard) did not find a significant difference between higher vs lower lithium levels.

In addition, 4 studies also looked at possible differences between the prevention of manic vs depressive recurrences. Only 1 study (Maj) suggested a lower threshold for the prevention of manic symptoms (around 0.45 mmol/L) than for depressive symptoms (around 0.60 mmol/L). Another study (Nolen) suggests the same threshold of 0.60 mmol/L for both the prevention of manic and depressive recurrences. The other 2 studies (Waters, Vestergaard) also found no difference in polarity of response and lithium levels.

Finally, 3 studies, addressed the association of lithium levels with adverse effects. Two studies, reported more side effects with levels 0.45‐0.60 and higher vs 0.30‐0‐45 (Maj) and with levels 0.80‐1.00 vs 0.40‐0.60 (Gelenberg), while the third study (Nolen) did not report different drop‐outs rates due to side effects between the 3 groups (lithium <0.60 mmol/L, lithium 0.60‐1.20 mmol/L and placebo).

### 3.5 Delphi survey

The Delphi survey was completed by 33 of the 50 (66.6%) Task Force members. Many statements received additional comments, most frequently that evidence regarding a statement or a specified response was lacking.

There was consensus on recommendations regarding timing of blood sampling to measure lithium serum levels during the day and on whether recommendations for optimal serum lithium levels should be the same with once daily dosing compared to twice daily dosing, as well as with different formulations of lithium medication (immediate release vs extended release).

There was a lack of consensus on the overall statement *“The higher the lithium serum level, the higher the likelihood of a preventative response*” (endorsed by only 54.5%) while several members commented that serum levels in the lower range (below 0.60 mmol/L) are associated with efficacy but in the higher range (above 1.00 mmol/L) with more adverse effects and toxicity. In addition, there was no consensus on the statement *“There is a minimum serum cut‐off level below which essentially no patients are likely to experience a preventative effect”* (endorsed by 72.7%) while there was a wide range of specified responses regarding for a specific cut‐off level (0.30, 0.40, 0.50, or 0.60 mmol/L, endorsed by 16.7, 41.7, 16.7, or 20.8%, respectively). In contrast, there was consensus on the statement *“There is a maximum lithium serum level that should ideally never be exceeded because of the risk of severe intolerance and/or intoxication”* (endorsed by 93.9%) but lack of agreement on a specific upper limit to recommend: most frequently supported limits being 1.00 and 1.20 mmol/L, endorsed by 35.5 and 38.7%, respectively.

The major recommendations including 7 statements endorsed by at least 80% of the members (consensus) are summarized in Table 5.

**Table 5:** Recommendations from the Delphi survey regarding lithium serum levels in the maintenance treatment of bipolar disorder endorsed by at least 80% (consensus) of the participating members of the task force, or by less than 80% (no consensus)

Domain	Recommendation	
Timing of blood sampling to measure lithium serum levels	* Consensus*	
	1	With twice daily dosing: sampling should be in the morning 12 ± 1 hours after intake of the (last) evening dose and before the morning dose (endorsed by 93.5%)
	2	With once daily dosing in the evening: sampling should be in the morning, 12 + 1 hours after intake of the (single) evening dose (endorsed by 93.5%)
	* No consensus*	
	‐	With once daily dosing in the morning: sampling should be in the evening, 12 ± 1 hours after intake of the (single) morning dose (endorsed by 74.2%)
Once daily dosing vs twice daily dosing	* Consensus*	
	3	The recommendations for optimal serum lithium levels with once daily dosing of lithium should be the same as compared to twice daily dosing (endorsed by 90.9%)
Immediate release formula vs extended release formulations	* Consensus*	
	4	The recommendations for optimal serum lithium levels with immediate release formulations should be the same as compared to extended release formulations (endorsed by 93.9%)
Maximum lithium serum level	* Consensus*	
	5	There is a maximum serum lithium level that should ideally never be exceeded because of the risk of severe intolerance and/or intoxication (endorsed by 93.9%)
	* No consensus* – Ι On specific maximum level	
	‐	Most frequently mentioned: 1.00 mmol/L (endorsed by 35.5%) and 1.20 mmol/L (endorsed by 38.7%)
Recommendations for optimal serum lithium levels in different age groups	* Consensus*	
	6	*For adults (18‐65 y):* The standard serum level should be 0.60‐0.80 mmol/L with the option to reduce the level to 0.40‐0.60 mmol/L in case of poor tolerance or to increase the level to 0.80‐1.00 mmol/L in case of insufficient response (endorsed by 93.9)
	* No consensus*	
	‐	*For children (<12 y):* The standard level should be 0.60‐0.80 mmol/L, with the option to reduce the level to 0.40‐0.60 mmol/L in case of poor tolerance or to increase the level to 0.80‐1.00 mmol/L in case of insufficient response (endorsed by 57.6%)
	‐	*For adolescents (12‐17 y):* The standard level should be 0.60‐0.80 mmol/L, with the option to reduce the level to 0.40‐0.60 mmol/L in case of poor tolerance or to increase the level to 0.80‐1.00 mmol/L in case of insufficient response (endorsed by 75.8%)
	‐	*For the elderly (65‐79 y):* The standard serum lithium level should be 0.40 ‐ 0.60 mmol/L, with the option to increase to 0.60 ‐ 0.80 mmol/L or to even 0.80 −1.00 mmol/L in case of insufficient response (unless there are somatic contra‐indications and with close monitoring of emergent side effects) (endorsed by 63.6%)
	–	*For the elderly (80 y and older):* The standard serum lithium level should be 0.40 ‐ 0.60 mmol/L, with the option to increase to 0.60 ‐ 0.70 mmol/L (and not higher) in case of insufficient response (unless there are somatic contra‐indications and with close monitoring of emergent side effects) (endorsed by 48.5%)
	–	The standard serum lithium level should be 0.40 ‐ 0.60 mmol/L, with the option to increase to 0.60 ‐ 0.80 mmol/L (and not higher) in case of insufficient response (unless there are somatic contra‐indications and with close monitoring of emergent side effects) (endorsed by 48.5%)
Optimal lithium serum levels for the prevention of specific episodes	* Consensus*	
	7	The recommendations for the prevention of episodes of mania or depression with mixed features should be the same as the recommendations to prevent manic recurrences (endorsed by 87.9%)
	* No consensus*	
	‐	The recommendations for the prevention of manic recurrences should be the same as the recommendations to prevent depressive recurrences (endorsed by 66.7%)
	‐	The recommendations for the prevention of hypomanic recurrences should be the same as the recommendations to prevent manic recurrences (endorsed by 78.8%)
	‐	The recommendations for the prevention of subsyndromal depressive recurrences should be the same as the recommendations to prevent depressive recurrences (endorsed by 78.8%)

Regarding recommended standard lithium serum levels in the maintenance treatment there was only consensus on the recommendation for adults, but not for children <12 years, adolescents 12‐17 years, the elderly 65‐79 and the elderly ≥80 years. However, the most frequently endorsed minimum level was 0.40 mmol/L for all age groups, whereas the maximum level varied with age; 1.00 mmol/L for adults, children and adolescents and between 0.70 and 1.00 mmol/L for the elderly.

Finally, for the prevention of specific episodes there was consensus for recommending similar levels for the prevention of mixed episodes vs manic episodes (endorsed by 87.9%) but not for recommending similar levels for the prevention of manic vs depressive episodes and for the prevention of hypomanic or subthreshold depressive episodes vs manic and depressive episodes, respectively.

## 4 DISCUSSION

The key conclusion from this review (and the major limitation) is that there is a lack of well‐designed, double‐blind randomized dose finding (or serum level finding) studies assessing optimal lithium serum level in the maintenance treatment of BP. This conclusion is truly remarkable given that lithium has been available for the treatment of BP since it was licensed in the early 1970s. This paucity of data makes it difficult if not impossible to formulate confident evidence‐based recommendations for the dosing of lithium based on monitoring its serum level. Based on the initial inclusion criteria for our systematic review we were unable to identify any study that fulfilled all 6 original inclusion criteria for our review. Only after loosening these criteria, we identified 7 studies that addressed, at least to some extent, the questions we posed at the outset, and outlined in the introduction.

### 4.1 Selection of studies

A major issue in the selection of studies was that we wanted to compare similar groups of patients treated with lithium at different serum levels while the composition of the groups should not be influenced by channeling of nonresponding patients with good tolerance to higher lithium level groups and early responding patients or patients with low tolerance to lower lithium level groups. Therefore, we initially intended to select only studies with a randomized design (criterion 5) in which patients were assigned to different treatment regimens with lithium resulting in different a priori defined fixed lithium serum level ranges (criterion 6). Five studies (Jerram, Waters, Coppen, Gelenberg, Vestergaard) were randomized. The remaining 2 studies (Maj, Nolen) were not randomized, but it is likely that the composition of the different lithium groups in these studies was not seriously affected by channeling. In the Maj study the patients were allocated in an open way to 4 different groups at baseline, while in the Nolen study due to an organizational failure (see above) a large group of patients remained below the predefined minimum level of 0.60 mmol/L, resulting in the possibility to compare 2 groups below or above this level.

Nevertheless, prior to start of follow‐up some channeling may have occurred in the pre‐randomization open treatment phase in which all patients (Jerram, Waters, Coppen, Gelenberg) or possibly some patients (Vestergaard) were already using lithium. In particular, if lithium had been given prophylactically with at least some success regarding efficacy and tolerability (Jerram, Waters, Coppen) the probability that relevant channeling with regard to the topic of the trial, namely the prophylactic efficacy of lithium, may have occurred, cannot be dismissed. Similarly, an unknown number of patients in the Maj study were already using lithium prior to allocation to one of the groups/start of follow‐up. This may have resulted in these studies in some selection bias: of poorer responding and better tolerating patients in the groups with higher lithium serum levels. In fact, the only study in which we can be confident that channeling did not happen regarding lithium levels prior to start of follow‐up, is the (nonrandomized) study by Nolen, as this is the only study where patients were not already using lithium at start of follow‐up.

An example of the excluded studies is the study by Tohen et al^31^ which compared in a randomized controlled, double‐blind design 2 groups of patients treated with olanzapine or lithium monotherapy after an open label phase in which they were treated for a manic episode with the combination of olanzapine and lithium. During the open label phase, lithium serum levels were targeted at 0.60‐1.20 mmol/L, depending on overall response to and tolerance of the combination. Only responders were randomized to the double blind maintenance phase (enriched sampling). However, some patients remained below 0.60 mmol/L, for instance when they did not tolerate lithium (combined with olanzapine) at the intended level. In a post‐hoc analysis of this study by Severus et al^35^ 3 subgroups were compared within the group randomized to continuing lithium at the same level as in the open treatment phase (<0.60, 0.60‐0.79 and ≥0.80 mmol/L). Although the original study protocol (31) prevented channeling in the double blind phase, channeling could have occurred during the open label phase as some patient had levels lower than 0.60 mmol/L and no new groups were constructed at the start of follow‐up in the original study. Thus, the design differed from the Maj study that did not include an acute phase while most patients were allocated open label to different lithium levels from the start of treatment with lithium, and from the Nolen study (see above). However, as the post‐hoc analysis (35) used proportional hazards Cox regression models and marginal structural models (MSMs) controlling for, among other variables, olanzapine and lithium dose at randomization as well as variables related to the disease severity and index episode features at baseline as well as variables related to the previous course of illness, the significance of the results of this post‐hoc analysis may in fact be comparable to those of a randomized controlled trial.

### 4.2 What is the optimal serum level for lithium in the maintenance treatment of bipolar disorder?

The main finding of our review is that 4 of the 7 included studies reported significantly fewer recurrences with lithium levels 0.80‐1.40 mmol/L vs 0.30‐0.80 (Waters); 0.61‐0.90 vs 030‐0.45 (Maj); 0.80‐1.00 vs 0.40‐0.60 (Gelenberg); and 0.60‐1.00 vs <0.60 mmol/L (Nolen). In addition, the latter study found superiority of 0.60‐1.20 vs placebo but not of <0.60 vs placebo. Taken together, these studies suggest better efficacy being associated with lithium levels in a therapeutic range with a lower threshold around 0.45/0.60 and up to 0.80/1.00 mmol/L. The other 3 studies (Jerram, Coppen, Vestergaard) did not find a significant difference between higher vs lower lithium levels.

There is also some additional information from some of the above studies. Hullin^40^ performed a further second year follow‐up of Jerram study of the combined group patients (n = 68) with BP or UP. In this second year 5/28 patients (17.8%) with lithium levels of 0.60‐1.00 mmol/L and 4/27 patients (14.8%) of those with levels between 0.40 and 0.59 had a recurrence. In contrast, in the group maintained on lithium levels between 0.25 and 0.39 mmol/L, 8/13 patients (61.5%) had a recurrence. Despite that no separate results were presented for the BP sample, this finding suggests that the cutoff for an effective lithium level may be around 0.40 mmol/L. In contrast however, a further subdivision of the 2 lithium groups in the Nolen study revealed that the Hazard ratio's (HR) vs placebo were not significant for lithium <0.40 mmol/L (HR 0.66; 95% CI: 0.42‐1.03) and 0.40 to <0.60 mmol/L (HR 1.05; CI: 0.67‐1.63) while they were significant for lithium 0.60 to <0.80 (HR 0.35; CI: O.23‐0.52) and 0.80 to <1.00 (HR 0.24; CI: 0.14‐0.42), but not for lithium 1.00‐1.20 (HR 0.50; CI: 0.21‐1.22). These findings suggest that the cutoff is more likely to be around 0.60 mmol/L than around 0.40 mmol/L. In general, while lithium levels <0.60 mmol/L appear not as effective as levels >0.60 mmol/L, there may be some patients for whom levels between 0.40 and 0.60 mmol/L are effective. Therefore, this may be an option for those who do not tolerate levels >0.60 mmol/L as well as for patients with impaired renal function or with increased risk of intoxication, for example, in the elderly (see below). In addition, levels <0.40 mmol/L do not seem to work at all. Therefore, there is no rationale for prescribing lithium at levels below 0.40 mmol/L.

Finally, the Nolen study also suggests no big additional benefit of levels 0.80 to <1.0 mmol/L over 0.60 to <0.85 mmol/L, which is in line with the Maj study which did not find a difference between levels 0.76‐0.90 mmol/L and 0.61‐0.75 mmol/L. Although we should recognize the possibility of a Type 2 error, for example, that with larger numbers a difference may have been found, these studies suggest an upper effective cutoff around 0.80 mmol/L. Nevertheless, again there may be some patients for whom higher levels up to 1.00 mmol/L are more effective and well tolerated. In addition, however, there are no data supporting the notion that going beyond 1.00 mmol/L is associated with any further benefit, possibly with exception of its use in the treatment of acute manic episodes (not further being discussed in this paper). In addition, the upper limit of 0.80 or 1.00 mmol/L is of course also defined by the increased risk of side effects and toxicity at higher lithium serum levels.^41^

**Figure 2:**
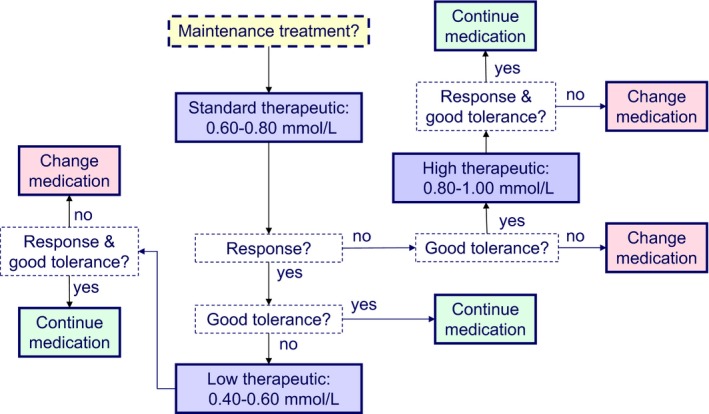
Recommendation for optimal lithium serum levels in the maintenance treatment of bipolar disorder: endorsed in consensus by the Task Force members for adults (18‐65 y) and by the majority of the members for children (<12 y) and adolescents (12‐17 y) [Colour figure can be viewed at wileyonlinelibrary.com]

Taking all these above data into account, they do support the outcome of the Delphi survey: that for adults (18‐65 years) the standard therapeutic lithium serum level in the maintenance treatment of bipolar disorder should be 0.60‐0.80 mmol/L with the option to reduce the level to the low therapeutic level of 0.40‐0.60 mmol/L in case of sufficient response but poor tolerance or to increase the level to the high therapeutic level of 0.80‐1.00 mmol/L in case of insufficient response and good tolerance (Figure 2). The recommendation for the standard therapeutic level of 0.60‐0.80 mmol/L is in line with the NICE guideline,^2^ the RANZCP guideline,^3^ the BAP guideline^5^ and the “Lithiumeter 2.0”.^16^

### 4.3 What is the best timing of blood sampling?

No study was found that addressed this question but most of the included studies followed the generally accepted recommendation to sample blood 12 ± 1 hours after the last (ie evening) intake. There was also consensus on this practice among the Task Force members for patients taking lithium twice daily or taking lithium once daily in the evening. However, there was no consensus on whether to sample in the evening or in the next morning for patients taking lithium once daily in the morning, a dosing schedule probably hardly applied in clinical practice.

### 4.4 Should the recommendations be different for dosing once daily vs twice daily and for immediate release vs extended release formulations?

There is pharmacogenetic evidence that a total daily dose of lithium given once daily results in a 10%‐15% higher 12 ± 1 hours trough level than when given twice daily.^42^ Nevertheless, there was consensus among the Task Force members that one could follow the same recommendations for optimal lithium serum levels for both dosing strategies. Similarly, the members also agreed that the recommendations should be the same for different formulations (immediate release vs extended release).

An important clinical implication of this approach is that the relative small changes in 12 ± 1 hours trough levels caused by changes in dosing schedule or lithium formulation, should not necessarily lead to adjustments of the aimed lithium levels. In the absence of solid evidence to support an alternative approach, this simple pragmatic approach is reasonable and also compatible with the likely intra‐individual variations that inevitably occur with respect to subsequent lithium levels obtained with a stable dose. In this context, it should be born in mind that the average lithium level in a given individual with a constant renal function and a constant intake of fluid and sodium entirely depends on the total daily dose.^8^

### 4.5 Should the optimal serum level be different for the prevention of manic (and mixed) episodes compared to depressive episodes?

While lithium is effective in the prevention of both manic and depressive episodes^43^ there is the question whether same lithium levels should be applied. The 4 studies (Waters, Maj, Vestergaard, Nolen) that addressed this issue did not find evidence that lithium serum levels for the prevention of specific episodes should be different than for other episodes. In the Delphi survey there was only consensus that the recommendations for the prevention of mixed episodes should be the same as for the prevention of manic episodes. The recommendations for other comparisons (ie manic vs depressive episodes; hypomanic vs manic episodes; subsyndromal depressive vs depressive episodes) did not result in consensus, although the majority of members supported the statements that the levels should be the same for the prevention of all types of episodes.

### 4.6 Should recommendations take ethnicity or gender into consideration?

We did not find any study addressing these topics in relation to optimal lithium serum levels. Therefore, we also did not formulate recommendations on these issues.

A specific group concerns pregnant women. Pregnant women can be treated with lithium, but the recommendations in this paper are not applicable for them. For more specific information, we refer to a recent paper by Wesseloo et al.^44^

### 4.7 Should recommendations for children and adolescents or for elderly patients be different from the recommendations for (younger) adults?

Two of the selected studies (Coppen, Vestergaard) did not provide information on whether they had included patients below 18 years or above 65 years and 2 other studies did not include patients below 18 years or above 65 years (Waters, Maj). The remaining 3 studies did only include patients above 18 years including elderly patients, but did not provide separate information on the outcome of the elderly patients. Thus, we can conclude that evidence on optimal lithium serum levels in children, adolescents and the elderly (see also Ref. 38) is lacking.

**Figure 3:**
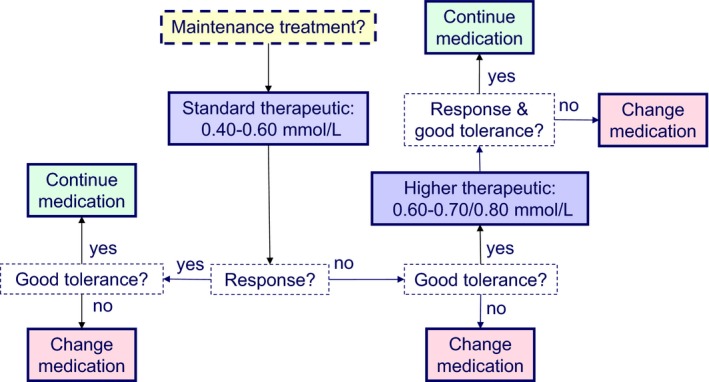
Recommendation for optimal lithium serum levels in the maintenance treatment of bipolar disorder in elderly patients, endorsed by the majority of the Task Force members for elderly patients (65 y and older) [Colour figure can be viewed at wileyonlinelibrary.com]

This is also reflected by outcome of the Delphi survey regarding the endorsement of the statements on optimal lithium serum levels in the various age groups of which only the statement on adults (18‐65 years) was endorsed by at least 80% (in consensus) of the Task Force members. However, for all age groups there was consensus that the minimum effective lithium level for all age groups should be 0.40 mmol/L, while in adults the standard therapeutic level should be between 0.60 and 0.80 mmol/L within a broader range of 0.40‐1.00 (Figure 2). For children (<12 years) and adolescents (12‐17 years) there was no consensus, but the majority of the Task Force members endorsed the same recommendation as for adults (see also Figure 2). For the elderly there was also no consensus. However, the majority of the members endorsed the recommendation that the standard therapeutic level should be 0.40‐0.60 mmol/L, with the option to go to maximally 0.70 of 0.80 mmol/L in elderly 65‐79 years and to maximally 0.7 mmol/L in elderly 80 years and older (Figure 3), which is in line with the recommendations on the use of lithium in the elderly from the ISBD Task Force on Older Adults with Bipolar Disorder: that although *“the balance between lithium toxicity and efficacy has not been studied in older patients and the recommendations are mainly based on clinical judgement and fear of drug‐related adverse events”*,^38^ the upper limit should be lower than in younger adults.^45^

### 4.8 Practical considerations regarding lithium level monitoring

Due to lithium's low therapeutic index, its serum levels should always be monitored during treatment, at least when treatment is initiated, after each dose increase and every 3‐6 months thereafter. The blood samples for measuring the lithium level must be drawn at steady state occurring after 5‐6 times the half‐life of lithium, which is approximately 24 hours in the nonelderly with normal renal function. Thus, the lithium level should be measured after 5‐6 days at constant daily dosing. In the elderly or in patients with impaired renal function, the time to steady state increases, implying that the lithium level should also be measured after 10‐12 days. In individual patients treated with a given schedule with a given lithium formulation, at steady state, the ratio of daily lithium dose, and the 12 hours lithium level is constant. An additional requirement for safe use of lithium is to monitor renal function at baseline and also every 3‐6 months.

Finally, it should be taken into account that subjects with a relatively high renal function, in particular young men, may have an unexpectedly low 12‐hours lithium level despite a relatively high daily lithium dose due to relative large variations in lithium levels over the day. If the dose is increased to obtain a lithium level within the recommended range, such patients may become intoxicated.^46^

## 5 CONCLUSION

Based on our review and the responses obtained in our Delphi survey we recommend that the standard serum level in the maintenance treatment of adults with BD should be 0.60‐0.80 mmol/L with the option to reduce the level to 0.40‐0.60 mmol/L in case of sufficient response but poor tolerance, or to increase the level to 0.80‐1.00 mmol/L in case of insufficient response and good tolerance. For children and adolescents the majority of the Task Force members endorsed the same recommendation, while for the elderly the majority endorsed a more conservative approach: usually 0.40‐0.60 mmol/L, with the option to go to maximally 0.70 or 0.80 mmol/L at ages 65‐79 years, and to maximally 0.70 mmol/L over age 80 years. Sampling should be in the morning 12 ± 1 hours after intake of the evening dose. Tentative recommendations for optimal serum lithium levels should be the same with once and twice daily dosing, and with immediate release formulations compared to extended release formulations.

While the above recommendations reflect the available evidence, more high‐quality research is still needed to provide more refined advices.

## CONFLICT OF INTEREST

The following authors reported a potential conflict of interest: Dr Licht has received research grant from Glaxo Smith Kline, honoraria for lecturing from Pfizer, Glaxo Smith Kline, Eli Lilly, Astra‐Zeneca, Bristol‐Myers Squibb, Janssen Cilag, Lundbeck, Otsuka, Servier, and honoraria from advisory board activity from Glaxo Smith Kline, Eli Lilly, Astra‐Zeneca, Bristol‐Myers Squibb, Janssen Cilag, and Sunovion. Dr Nielsen has received research grants from H. Lundbeck and Otsuka Pharmaceuticals for clinical trials, received speaking fees from Bristol‐Myers Squibb, Astra Zeneca, Janssen & Cilag, Lundbeck, Servier, Otsuka Pharmaceuticals, and Eli Lilly and has acted as advisor to Astra Zeneca, Eli Lilly, Lundbeck, Otsuka Pharmaceuticals, Takeda, and Medivir. Dr Severus received speaker honoraria from Servier, Roche and Aristo in the past 3 years. Dr Vieta has received grants and served as consultant, advisor or CME speaker for the following entities: AB‐Biotics, Abbott, Allergan, Angelini, AstraZeneca, Bristol‐Myers Squibb, Dainippon Sumitomo Pharma, Farmindustria, Ferrer, Forest Research Institute, Gedeon Richter, Glaxo‐Smith‐Kline, Janssen, Lundbeck, Otsuka, Pfizer, Roche, SAGE, Sanofi‐Aventis, Servier, Shire, Sunovion, Takeda, the Brain and Behaviour Foundation, the Spanish Ministry of Science and Innovation (CIBERSAM), EU Horizon 2020, and the Stanley Medical Research Institute. Dr Zarate is a full‐time U.S government employee and is an inventor on several patent and patent applications related to ketamine and ketamine metabolites that have all been assigned to the US Government. He will share a percentage of any royalties that may be received by the Government in accordance to NIH policy.

## MEMBERS OF THE ISBD/IGSLI TASK FORCE ON THE TREATMENT WITH LITHIUM WHO COMPLETED THE DELPHI SURVEY AND APPROVED THE PAPER

M. Alda, Dalhousie University, Halifax, Canada.

M. Bauer, University Hospital Carl Gustav Carus, TU Dresden, Dresden, Germany.

A. Berghöfer, Charité – Universitätsmedizin, Berlin, Germany.

M. Berk, Deakin University, Geelong, Australia.

A. Bocchetta, University of Cagliari, Cagliari, Italy.

C. V. Calkin, Dalhousie University, Halifax, NS, Canada.

A. Duffy, Queens's University, Kingston, Canada.

K. N. Fountoulakis, Aristotle University of Thessaloniki, Thessaloniki, Greece.

A. Gonzalez‐Pinto, CIBERSAM‐BioAraba Research Institute, Vitoria, Spain.

T. Hajek, Dalhousie University, Halifax, NS, Canada.

L. V. Kessing, Psychiatric Center Copenhagen, Copenhagen, Denmark.

C. López‐Jaramillo, Universidad de Antioquia, Medellin, Colombia.

R. Machado‐Vieira University of Texas Health Science Center, Houston,TX, USA.

R. M. Post, Bipolar Collaborative Network, Bethesda, MD, USA.

J. K. Rybakowski, Poznan University of Medical Sciences, Poznan, Poland.

C. Simhandl, Sigmund Freud Privat Universität, Wien, Austria.

J. C. Soares, University of Texas Health Science Center, Houston, TX, USA.

L. Tondo, Lucio Bini Mood Centers, Cagliari and Rome, Italy.

## References

[1] Grunze H , Vieta E , Goodwin GM , et al. The world federation of societies of biological psychiatry (WFSBP) guidelines for the biological treatment of bipolar disorders: update 2012 on the long‐term treatment of bipolar disorder. World J Biol Psychiatry. 2013;14(3):154‐219. 23480132 10.3109/15622975.2013.770551

[2] NICE‐2014 . Bipolar Disorder: Assessment and Management (NICE2014). Available at: https://www.nice.org.uk/guidance/cg185. Accessed January 31, 2018.

[3] Malhi GS , Bassett D , Boyce P , et al. Royal Australian and New Zealand College of Psychiatrists clinical practice guidelines for mood disorders. Aust N Z J Psychiatry. 2015;2015(49):1087‐1206. 10.1177/0004867415617657 26643054

[4] Kupka R , Goossens P , Van Bendegem M , et al. Multidisciplinaire richtlijn bipolaire stoornissen, derde herziene versie. Utrecht, the Netherlands: Trimbos Instituut; 2015 Available at: https://www.nvvp.net/stream/richtlijn-bipolaire-stoornissen-2015.

[5] Goodwin GM , Haddad PM , Ferrier IN , et al. Evidence‐based guidelines for treating bipolar disorder: revised third edition recommendations from the British Association for Psychopharmacology. J Psychopharmacol. 2016;30(6):495‐553. 26979387 10.1177/0269881116636545 PMC4922419

[6] Fountoulakis KN , Yatham L , Grunze H , et al. The International College of Neuro‐Psychopharmacology (CINP) treatment guidelines for bipolar disorder in adults (CINP‐BD‐2017), part 2: review, grading of the evidence, and a precise algorithm. Int J Neuropsychopharmacol. 2017;20:121‐179. 27816941 10.1093/ijnp/pyw100 PMC5409012

[7] Yatham LN , Kennedy SH , Parikh SV , et al. Canadian Network for Mood and Anxiety Treatments (CANMAT) and International Society for Bipolar Disorders (ISBD) 2018 guidelines for the management of patients with bipolar disorder. Bipolar Disord. 2018;20:97‐170. 29536616 10.1111/bdi.12609 PMC5947163

[8] Licht RW . Lithium: still a major option in the management of bipolar disorder. CNS Neurosci Ther. 2012;18:219‐226. 22070642 10.1111/j.1755-5949.2011.00260.x PMC6493602

[9] Sashidharan SP . The relationship between serum lithium levels and clinical response. Ther Drug Monit. 1982;4:249‐264. 6753253 10.1097/00007691-198208000-00002

[10] Severus WE , Kleindienst N , Seemüller F , Frangou S , Möller HJ , Greil W . What is the optimal serum lithium level in the long‐term treatment of bipolar disorder–a review? Bipolar Disord. 2008;10:231‐237. 18271901 10.1111/j.1399-5618.2007.00475.x

[11] Malhi GS , Gessler D , Outhred T . The use of lithium for the treatment of bipolar disorder: recommendations from clinical practice guidelines. J Affect Disord. 2017;217:266‐280. 28437764 10.1016/j.jad.2017.03.052

[12] Wijeratne C , Draper B . Reformulation of current recommendations for target serum lithium concentration according to clinical indication, age and physical comorbidity. Aust N Z J Psychiatry. 2011;45:1026‐1032. 21961481 10.3109/00048674.2011.610296

[13] Hopkins HS , Gelenberg AJ . Serum lithium levels and the outcome of maintenance therapy of bipolar disorder. Bipolar Disord. 2000;2(3 Pt 1):174‐179. 11256684 10.1034/j.1399-5618.2000.020304.x

[14] McIntyre RS , Mancini DA , Parikh S , Kennedy SH . Lithium revisited. Can J Psychiatry. 2001;46:322‐327. 11387787 10.1177/070674370104600402

[15] Sproule B . Lithium in bipolar disorder: can drug concentrations predict therapeutic effect? Clin Pharmacokinet. 2002;41:639‐660. 12126457 10.2165/00003088-200241090-00002

[16] Malhi GS , Gershon S , Outhred T . Lithiumeter: version 2.0. Bipolar Disord. 2016;18:631‐641. 28063207 10.1111/bdi.12455

[17] Leufkens HG , Urquhart J . Variability in patterns of drug usage. J Pharm Pharmacol. 1994;46(Suppl 1):433‐437. 8064562

[18] Tohen M , Waternaux CM , Tsuang MT . Outcome in Mania. A 4‐year prospective follow‐up of 75 patients utilizing survival analysis. Arch Gen Psychiatry. 1990;47:1106‐1111. 2244795 10.1001/archpsyc.1990.01810240026005

[19] Jadad AR , Moore RA , Carroll D , et al. Assessing the quality of reports of randomized clinical trials: is blinding necessary? Control Clin Trials. 1996;17:11‐12. 10.1016/0197-2456(95)00134-4 8721797

[20] Jones J , Hunter D . Consensus methods for medical and health services research. BMJ. 1995;311:376‐380. 7640549 10.1136/bmj.311.7001.376 PMC2550437

[21] Jerram TC , McDonald R . Plasma lithium control with particular reference to minimum effective levels In: Johnson FN, Johnson S, eds. Lithium in medical practice. Lancaster, UK: MTP Press Ltd.; 1978:407‐413.

[22] Waters B , Lapierre Y , Gagnon A , et al. Determination of the optimal concentration of lithium for the prophylaxis of manic‐depressive disorder. Biol Psychiatry. 1982;17:1323‐1329. 6817830

[23] Coppen A , Abou‐Saleh M , Milln P , Bailey J , Wood K . Decreasing lithium dosage reduces morbidity and side‐effects during prophylaxis. J Affect Disord. 1983;5:353‐362. 6229567 10.1016/0165-0327(83)90026-5

[24] Maj M , Starace F , Nolfe G , Kemali D . Minimum plasma lithium levels required for effective prophylaxis in DSM III bipolar disorder: a prospective study. Pharmacopsychiatry. 1986;19:420‐423. 3797469 10.1055/s-2007-1017280

[25] Gelenberg AJ , Kane JM , Keller MB , et al. Comparison of standard and low serum levels of lithium for maintenance treatment of bipolar disorder. N Engl J Med. 1989;321:1489‐1493. 2811970 10.1056/NEJM198911303212201

[26] Vestergaard P , Licht RW , Brodersen A , et al. Outcome of lithium prophylaxis: a prospective follow‐up of affective disorder patients assigned to high and low serum lithium levels. Acta Psychiatr Scand. 1998;98:310‐315. 9821453 10.1111/j.1600-0447.1998.tb10089.x

[27] Nolen WA , Weisler RH . The association of the effect of lithium in the maintenance treatment of bipolar disorder with lithium plasma levels: a post hoc analysis of a double‐blind study comparing switching to lithium or placebo in patients who responded to quetiapine (Trial 144). Bipolar Disord. 2013;15:100‐109. 23228201 10.1111/bdi.12027

[28] Sashidharan SP , McGuire RJ , Glen AI . Plasma lithium levels and therapeutic outcome in the prophylaxis of affective disorders: a retrospective study. Br J Psychiatry. 1982;140:619‐622. 7104552 10.1192/bjp.140.6.619

[29] Goodnick PJ , Fieve RR . Plasma lithium level and interepisode functioning in bipolar disorder. Am J Psychiatry. 1985;142:761‐762. 4003601 10.1176/ajp.142.6.761

[30] Kleindienst N , Severus WE , Möller HJ , Greil W . Is polarity of recurrence related to serum lithium level in patients with bipolar disorder? Eur Arch Psychiatry Clin Neurosci. 2005;255:72‐74. 15711896 10.1007/s00406-005-0574-x

[31] Tohen M , Greil W , Calabrese JR , et al. Olanzapine versus lithium in the maintenance treatment of bipolar disorder: a 12‐month, randomized, double‐blind, controlled clinical trial. Am J Psychiatry. 2005;162:1281‐1290. 15994710 10.1176/appi.ajp.162.7.1281

[32] Keller MB , Lavori PW , Kane JM , et al. Subsyndromal symptoms in bipolar disorder. A comparison of standard and low serum levels of lithium. Arch Gen Psychiatry. 1992;49:371‐376. 1586272 10.1001/archpsyc.1992.01820050035005

[33] Solomon DA , Ristow WR , Keller MB , et al. Serum lithium levels and psychosocial function in patients with bipolar I disorder. Am J Psychiatry. 1996;153:1301‐1307. 8831438 10.1176/ajp.153.10.1301

[34] Perlis RH , Sachs GS , Lafer B , et al. Effect of abrupt change from standard to low serum levels of lithium: a reanalysis of double‐blind lithium maintenance data. Am J Psychiatry. 2002;159:1155‐1159. 12091193 10.1176/appi.ajp.159.7.1155

[35] Severus WE , Lipkovich IA , Licht RW . In search of optimal lithium levels and olanzapine doses in the long‐term treatment of bipolar I disorder. A post‐hoc analysis of the maintenance study by Tohen et al. 2005. Eur Psychiatry. 2010;25:443‐449. 20430594 10.1016/j.eurpsy.2009.10.009

[36] Severus WE , Kleindienst N , Evoniuk G , et al. Is the polarity of relapse/recurrence in bipolar‐I disorder patients related to serum lithium levels? Results from an empirical study. J Affect Disord. 2009;115:466‐470. 19019453 10.1016/j.jad.2008.10.009

[37] Severus WE , Grunze H , Kleindienst N , Frangou S , Moeller HJ . Is the prophylactic antidepressant efficacy of lithium in bipolar I disorder dependent on study design and lithium level? J Clin Psychopharmacol. 2005;25:457‐462. 16160621 10.1097/01.jcp.0000177550.13714.7a

[38] Dols A , Kessing LV , Strejilevich SA , et al. International Society for Bipolar Disorders Task Force for Older Adults with Bipolar Disorder . Do current national and international guidelines have specific recommendations for older adults with bipolar disorder? A brief report. Int J Geriatr Psychiatry. 2016;31:1295‐1300. 27442023 10.1002/gps.4534

[39] Weisler RH , Nolen WA , Neijber A , Hellqvist A , Paulsson B ; Trial 144 Study Investigators . Continuation of quetiapine versus switching to placebo or lithium for maintenance treatment of bipolar I disorder (Trial 144: a randomized controlled study). J Clin Psychiatry. 2011;72:1452‐1464. 22054050 10.4088/JCP.11m06878

[40] Hullin RP . Minimum serum lithium levels for effective prophylaxis In: Johnson FN, ed. Handbook of Lithium Therapy. Lancaster, UK: MTP Press Limited; 1980:243‐247.

[41] Wilting I , Heerdink ER , Mersch PP , Den Boer JA , Egberts AC , Nolen WA . Association between lithium serum level, mood state, and patient‐reported adverse drug reactions during long‐term lithium treatment: a naturalistic follow‐up study. Bipolar Disord. 2009;11:434‐440. 19500096 10.1111/j.1399-5618.2009.00699.x

[42] Amdisen A . Serum level monitoring and clinical pharmacokinetics of lithium. Clin Pharmacokinet. 1977;2:73‐92. 324690 10.2165/00003088-197702020-00001

[43] Severus E , Taylor MJ , Sauer C , et al. Lithium for prevention of mood episodes in bipolar disorders: systematic review and meta‐analysis. Int J Bipolar Disord. 2014;2:15. 25530932 10.1186/s40345-014-0015-8 PMC4272359

[44] Wesseloo R , Wierdsma AI , van Kamp IL , et al. Lithium dosing strategies during pregnancy and the postpartum period. Br J Psychiatry. 2017;211:31‐36. 28673946 10.1192/bjp.bp.116.192799 PMC5494438

[45] Shulman KI , Almeida OP , Herrmann N , et al. Delphi survey of maintenance lithium treatment in older adults with bipolar disorder: an ISBD task force report. Bipolar Disord. 2019;21(2):117‐123. 30375703 10.1111/bdi.12714 PMC6587471

[46] Amdisen A . Lithium neurotoxicity – the reliability of serum lithium measurements. Hum Psychopharmacol. 1990;5:281‐285.

