# Supplementary material for: What is the optimal serum level for lithium in the maintenance treatment of bipolar disorder? A systematic review and recommendations from the ISBD/IGSLI Task Force on treatment with lithium
Source: Bipolar Disord. 2019 Jun 20;21(5):394–409. doi: 10.1111/bdi.12805 (PMC6688930; doi:10.1111/bdi.12805)
Supplement: Supplementary file 1 [file BDI-21-394-s001.docx]

**Supplemental table 1:** Delphi Survey (Round 1)

***Please complete the survey and provide your comments, if any***

***Contact information***

First Name ........................

Last Name ........................

Country ........................

Email Address ........................

1. In the ***maintenance treatment of bipolar disorder*** is there a minimum serum lithium level below which essentially no patients are likely to experience a preventative effect?
   - **Statement**: There is a minimum serum cut-off level [ ] Agree [ ] Not agree
   - If you agree: **Choose one:**
     - Below 0.20 mmol/l [ ]
     - Below 0.30 mmol/l [ ]
     - Below 0.40 mmol/l [ ]
     - Below 0.50 mmol/l [ ]
     - Below 0.60 mmol/l [ ]
   - Comments (if any) ……………………………………………………………………………………………

…………………………………………………………………………………………………………………………

1. In the ***maintenance treatment of bipolar disorder*** is there a relationship between serum lithium level and the efficacy of lithium?
   - **Statement**: The higher the serum lithium level, the higher likelihood of a preventative response [ ] Agree [ ] Not agree
   - If you agree: **Choose one range:**
     - In the range of 0.20 – 0.8 mmol/l [ ]
     - In the range of 0.20 – 1.0 mmol/l [ ]
     - In the range of 0.20 – 1.2 mmol/l [ ]
     - In the range of 0.20 – 1.5 mmol/l [ ]
     - In the range of 0.30 – 0.8 mmol/l [ ]
     - In the range of 0.30 – 1.0 mmol/l [ ]
     - In the range of 0.30 – 1.2 mmol/l [ ]
     - In the range of 0.30 – 1.5 mmol/l [ ]
     - In the range of 0.40 – 0.8 mmol/l [ ]
     - In the range of 0.40 – 1.0 mmol/l [ ]
     - In the range of 0.40 – 1.2 mmol/l [ ]
     - In the range of 0.40 – 1.5 mmol/l [ ]
     - In the range of 0.50 – 0.8 mmol/l [ ]
     - In the range of 0.50 – 1.0 mmol/l [ ]
     - In the range of 0.50 – 1.2 mmol/l [ ]
     - In the range of 0.50 – 1.5 mmol/l [ ]
     - In the range of 0.60 – 0.8 mmol/l [ ]
     - In the range of 0.60 – 1.0 mmol/l [ ]
     - In the range of 0.60 – 1.2 mmol/l [ ]
     - In the range of 0.60 – 1.5 mmol/l [ ]
     - In the range of 0.70 – 0.8 mmol/l [ ]
     - In the range of 0.70 – 1.0 mmol/l [ ]
     - In the range of 0.70 – 1.2 mmol/l [ ]
     - In the range of 0.70 – 1.5 mmol/l [ ]
     - In the range of 0.80 – 1.0 mmol/l [ ]
     - In the range of 0.80 – 1.2 mmol/l [ ]
     - In the range of 0.80 – 1.5 mmol/l [ ]
     - Agree, but not in any of these ranges [ ]

Write in your range ................................

- - Comments (if any) ……………………………………………………………………………………………

…………………………………………………………………………………………………………………………

1. In the ***maintenance treatment of bipolar disorder*** is there a maximum serum lithium level that should ideally never be exceeded because of risk of severe intolerance and/or intoxication?
   - **Statement**: There is such a maximum level [ ] Agree [ ] Not agree
   - If you agree: **What is that maximum serum lithium level:**
     - Above 1.0 mmol/l [ ]
     - Above 1.1 mmol/l [ ]
     - Above 1.2mmol/l [ ]
     - Above 1.3 mmol/l [ ]
     - Above 1.4 mmol/l [ ]
     - Above 1.5 mmol/l [ ]
     - Above 1.5mmol/l [ ]
     - Above 1.6 mmol/l [ ]
     - Above 1.7 mmol/l [ ]
     - Above 1.8 mmol/l [ ]
     - Above 1.9mmol/l [ ]
     - Above 2.0 mmol/l [ ]
   - Comments (if any) ……………………………………………………………………………………………

…………………………………………………………………………………………………………………………

1. Are you aware of a standard schedule to measure lithium serum levels ***after commencing treatment***?
   - **Statement**: There is such a standard schedule [ ] Agree [ ] Not agree
   - If you agree:
     - *Given* ***normal*** *elimination / glomerular filtration rate*

**Choose one:**

- - - After 1 day [ ]
    - After 3 days [ ]
    - After 5 days [ ]
    - After 7-10 days [ ]
    - After 1, 3 and 5 days [ ]
    - After 3 and 5 days [ ]
    - After 1, 5 and 7-10 days [ ]
    - After 3, 5 and 7-10 days [ ]
    - After 1, 3, 5 and 7-10 days [ ]
  - Comments (if any) ……………………………………………………………………………………….…

……………………………………………………………………………………………………………………….

- - - *Given* ***slow*** *elimination / reduced glomerular filtration rate*

**Choose one:**

- - - After 3 days [ ]
    - After 5 days [ ]
    - After 7-10 days [ ]
    - After 3 and 7-10 days [ ]
    - After 5 and 7-10 days [ ]
    - After 3, 5 and 7-10 days [ ]
  - Comments (if any) ……………………………………………………………………………………………

…………………………………………………………………………………………………………………………

1. Is there a recommended ***timing of blood sampling*** to measure lithium serum levels during the day?
   - **Statement**: There is such a proper schedule [ ] Agree [ ] Not agree
   - If agreed:
   - *With* ***twice daily*** *dosing:*
     - **Statement**: Sampling should be in the morning, 12±1 hours after intake of the (last) evening dose [ ] Agree [ ] Not agree
     - Comments (if any) …………………………………………………………..................…………………………….……
   - *With* ***once daily*** *dosing in the* ***evening***:

**Statement**: Sampling should be in the morning, 12±1 hours after intake of the (single) evening dose [ ] Agree [ ] Not agree

- - - Comments (if any) …………………………………………………………..................…………………………….……
  - *With* ***once daily*** *dosing in the* ***morning****:*
    - **Statement**: Sampling should be in the morning, 24±1 hours after intake of the (single) morning dose [ ] Agree [ ] Not agree
    - Comments (if any) …………………………………………………………..................…………………………….……
    - **Statement**: Sampling should be in the evening, 12±1 hours after intake of the (single) morning dose [ ] Agree [ ] Not agree
    - Comments (if any) ……………………………………………………………………………………….…......................

1. In the ***maintenance treatment of bipolar disorder*** should the recommendations for optimal serum lithium levels with once daily dosing of lithium be the same compared to twice daily dosing?
   - **Statement**: The recommendations for serum lithium levels with once daily dosing of lithium should be the same compared to twice daily dosing

[ ] Agree [ ] Not agree

- - Comments (if any) ……………………………………………………………………………………….….

…………………………………………………………………………………………………………………………

1. In the ***maintenance treatment of bipolar disorder***s should the recommendations for optimal serum lithium levels with immediate release formula be the same compared to extended release formula?
   - **Statement**: The recommendations for serum lithium levels with immediate release formula of lithium should be the same compared to extended release formula [ ] Agree [ ] Not agree
   - Comments (if any) ……………………………………………………………………………………….…

……………………………………………………………………………………………………………………….

1. In the ***maintenance treatment of bipolar disorder*** what should be the optimal recommendation for serum lithium levels?

***Please respond to all four suggested recommendations below***

- - **Statement**: The recommended standard serum lithium level should be 0.60-0.80 mmol/l with the option **to reduce** the level to 0.40-0.60 mmol/l in case of poor tolerance **or to increase** the level to 0.80-1.00 mmol/l in case of insufficient response to the left [ ] Agree [ ] Not agree
  - Comments (if any) ……………………………………………………………………………………….…

……………………………………………………………………………………………………………………….

- - **Statement**: The recommended standard serum lithium level should be 0.40-0.60 mmol/l, with the option **to increase** the level to 0.60-0.80 mmol/l **or to even** 0.80-1.00 mmol/l in case of insufficient response

to the left [ ] Agree [ ] Not agree

- - Comments (if any) ……………………………………………………………………………………….…

……………………………………………………………………………………………………………………….

- - **Statement**: The recommended standard serum lithium level should be 0.80-1.00 mmol/l, with the option **to reduce** the level to 0.60-0.80 mmol/l **or to even** 0.40-0.60 mmol/l in case of poor tolerance [ ] Agree [ ] Not agree
  - Comments (if any) ……………………………………………………………………………………….…

……………………………………………………………………………………………………………………….

- - **Statement**: The recommended standard serum lithium level should be 0.60-0.80 mmol/l, with the option **to increase** the level to 0.80-1.00 mmol/l **or to even** 0.80-1.20 mmol/l in case of insufficient response [ ] Agree [ ] Not agree
  - Comments (if any) ……………………………………………………………………………………….…

……………………………………………………………………………………………………………………….

- - If not agreed with any of these four recommendations above:

Write in your recommendation: ............................................................

1. In the ***maintenance treatment of bipolar disorder*** should the recommendations for serum lithium levels to prevent manic recurrences be ***the same*** as the recommendations to prevent depressive recurrences?
   - **Statement**: The recommendations for the prevention of manic recurrences should be the same as the recommendations to prevent depressive recurrences [ ] Agree [ ] Not agree
   - Comments (if any) ……………………………………………………………………………………….…

…………………………………………………………………………………………………………….…………

1. In the ***maintenance treatment of bipolar disorder*** should the recommendations for serum lithium levels to prevent mixed recurrences (episodes of mania or depression with mixed features) be ***the same*** as the recommendations to prevent manic recurrences?
   - **Statement**: The recommendations for the prevention of mixed recurrences should be the same as the recommendations to prevent manic recurrences [ ] Agree [ ] Not agree
   - Comments (if any) ……………………………………………………………………………………….…

…………………………………………………………………………………………………………….…………

1. Forgotten In the ***maintenance treatment of bipolar disorder***s should the recommendations for serum lithium levels to prevent subsyndromal manic recurrences be ***the same*** as the recommendations to prevent manic recurrences?
   - **Statement**: The recommendations for the prevention of subsyndromal manic recurrences should be the same as the recommendations to prevent manic recurrences [ ] Agree [ ] Not agree
   - Comments (if any) ……………………………………………………………………………………….…

…………………………………………………………………………………………………………….…………

1. In the ***maintenance treatment of bipolar disorder*** should the recommendations for serum lithium levels to prevent subsyndromal depressive recurrences ***the same*** as from the recommendations to prevent depressive recurrences?
   - **Statement**: The recommendations for the prevention of subsyndromal depressive recurrences should be the same as the recommendations to prevent depressive recurrences [ ] Agree [ ] Not agree
   - Comments (if any) ……………………………………………………………………………………….…

…………………………………………………………………………………………………………….…………

1. In the ***maintenance treatment of bipolar disorder***s should the recommendations for children <12 years be ***the same*** as the recommendations for adults?
   - **Statement**: The recommended serum levels for children <12 years should be the same as for adults [ ] Agree [ ] Not agree
   - Comments (if any) ……………………………………………………………………………………….…

……………………………………………………………………………………………………………………….

***Please respond to all two suggested recommendations below***

- - **Statement**: The recommended standard serum lithium level for children <12 years should be 0.60-0.80 mmol/l, with the option **to reduce** the level to 0.40-0.60 mmol/l in case of poor tolerance **or to increase** the level to 0.80-1.00 mmol/l in case of insufficient response [ ] Agree [ ] Not agree
  - Comments (if any) ……………………………………………………………………………………….…

……………………………………………………………………………………………………………………….

- - **Statement**: The recommended standard serum lithium level for children <12 years should be 0.40-0.60 mmol/l, with the option **to increase** the level to 0.60-0.80 mmol/l **or to even** 0.80-1.00 mmol/l in case of insufficient response [ ] Agree [ ] Not agree
  - Comments (if any) ……………………………………………………………………………………….…

…………………………………………………………………………………………………………….…………

- - If not agreed with any of these two recommendations above:

Write in your recommendation: ............................................................

1. In the ***maintenance treatment of bipolar disorder***s should the recommendations for adolescents 12-18 years be ***the same*** as the recommendations for adults?
   - **Statement**: The recommended serum levels for adolescents 12-18 years should be the same as for adults [ ] Agree [ ] Not agree
   - Comments (if any) ……………………………………………………………………………………….…

……………………………………………………………………………………………………………………….

***Please respond to all two suggested recommendations below***

- - **Statement**: The recommended standard serum lithium level for adolescents 12-18 years should be 0.60-0.80 mmol/l, with the option to reduce the level to 0.40-0.60 mmol/l in case of poor tolerance **or to increase** the level to 0.80-1.00 mmol/l in case of insufficient response [ ] Agree [ ] Not agree
  - Comments (if any) ……………………………………………………………………………………….…

……………………………………………………………………………………………………………………….

- - **Statement**: The recommended standard serum lithium level for adolescents 12-18 years should be 0.40-0.60 mmol/l, with the option **to increase** the level to 0.60-0.80 mmol/l **or to even** 0.80-1.00 mmol/l in case of insufficient response [ ] Agree [ ] Not agree
  - Comments (if any) ……………………………………………………………………………………….…

…………………………………………………………………………………………………………….…………

- - If not agreed with any of these two recommendations above:

Write in your recommendation: ............................................................

1. In the ***maintenance treatment of bipolar disorder***s should the recommendations for serum lithium levels in the maintenance treatment of bipolar disorder in the elderly 65-80 years be ***the same*** as the recommendations for (younger) adults?
   - **Statement**: The recommended standard serum levels for the elderly **60-79 years** should be the same as for non-elderly adults (unless there are somatic contra-indications and with close monitoring of emergent side effects) [ ] Agree [ ] Not agree
   - Comments (if any) ……………………………………………………………………………………….…

……………………………………………………………………………………………………………………….

***Please respond to all two suggested recommendations below***

- - **Statement**: The recommended standard serum lithium level for the elderly 60-79 years should be 0.40-0.60 mmol/l, with the option ***to increase*** the level to 0.60-0.80 mmol/l **or to even** 0.80-1.00 mmol/l in case of insufficient response (unless there are somatic contra-indications and with close monitoring of emergent side effects) [ ] Agree [ ] Not agree
  - Comments (if any) ……………………………………………………………………………………….…

……………………………………………………………………………………………………………………….

- - **Statement**: The recommended standard serum lithium level for the elderly 60-79 years should be 0.60-0.80 mmol/l, with the option **to reduce** the level to 0.40-0.60 mmol/l in case of poor tolerance **or to increase** the level to 0.80-1.00 mmol/l in case of insufficient response (unless there are somatic contra-indications and with close monitoring of emergent side effects)

[ ] Agree [ ] Not agree

- - Comments (if any) ……………………………………………………………………………………….…

……………………………………………………………………………………………………………………….

- - If not agreed with any of these two recommendations above:

Write in your recommendation: ............................................................

1. In the ***maintenance treatment of bipolar disorder***s should the recommendations for serum lithium levels in the maintenance treatment of bipolar disorder in the elderly 80 years and older be ***the same*** as the recommendations for (younger) adults?
   - **Statement**: The recommended standard serum levels for the elderly 80 years and older should be the same as for non-elderly adults (unless there are somatic contra-indications and with close monitoring of emergent side effects) to the left [ ] Agree [ ] Not agree
   - Comments (if any) ……………………………………………………………………………………….…

……………………………………………………………………………………………………………………….

***Please respond to all five suggested recommendations below***

- - **Statement**: The recommended standard serum lithium level for the elderly 80 years and older should be 0.40-0.60 mmol/l, with the option **to increase** the level to 0.60-0.70 mmol/l **(and not higher)** in case of insufficient response (unless there are somatic contra-indications and with close monitoring of emergent side effects) [ ] Agree [ ] Not agree
  - Comments (if any) ……………………………………………………………………………………….…

……………………………………………………………………………………………………………………….

- - **Statement**: The recommended standard serum lithium level for the elderly 80 years and older should be 0.40-0.60 mmol/l, with the option **to increase** the level to 0.60-0.70 mmol/l **or to even** 0.70-1.00 mmol/l in case of insufficient response (unless there are somatic contra-indications and with close monitoring of emergent side effects) [ ] Agree [ ] Not agree
  - Comments (if any) ……………………………………………………………………………………….…

……………………………………………………………………………………………………………………….

- - **Statement**: The recommended standard serum lithium level for the elderly 80 years and older should be 0.40-0.60 mmol/l, with the option **to increase** the level to 0.60-0.80 mmol/l **(and not higher)** in case of insufficient response (unless there are somatic contra-indications and with close monitoring of emergent side effects) [ ] Agree [ ] Not agree
  - Comments (if any) ……………………………………………………………………………………….…

……………………………………………………………………………………………………………………….

- - **Statement**: The recommended standard serum lithium level for the elderly 80 years and older should be 0.40-0.60 mmol/l, with the option **to increase** the level to 0.60-0.80 mmol/l **or to even** 0.80-1.00 mmol/l in case of insufficient response (unless there are somatic contra-indications and with close monitoring of emergent side effects) [ ] Agree [ ] Not agree
  - Comments (if any) ……………………………………………………………………………………….…

……………………………………………………………………………………………………………………….

- - **Statement**: The recommended standard serum lithium level for the elderly 80 years and older should be 0.60-0.80 mmol/l, with the option **to reduce** the level to 0.40-0.60 mmol/l in case of poor tolerance **or to increase** the level to 0.80-1.00 mmol/l in case of insufficient response (unless there are somatic contra-indications and with close monitoring of emergent side effects) [ ] Agree [ ] Not agree
  - Comments (if any) ……………………………………………………………………………………….…

…………………………………………………………………………………………………………….…………

- - If not agreed with any of these two recommendations above:

Write in your recommendation: ............................................................
